# The Importance of LDL and Cholesterol Metabolism for Prostate Epithelial Cell Growth

**DOI:** 10.1371/journal.pone.0039445

**Published:** 2012-06-27

**Authors:** Teemu J. Murtola, Heimo Syvälä, Pasi Pennanen, Merja Bläuer, Tiina Solakivi, Timo Ylikomi, Teuvo L. J. Tammela

**Affiliations:** 1 School of Medicine, University of Tampere, Tampere, Finland; 2 Department of Urology, Tampere University Hospital, Tampere, Finland; 3 Department of Anatomy, School of Medicine, University of Tampere, Tampere, Finland; 4 Department of Cell Biology, School of Medicine, University of Tampere, Tampere, Finland; 5 Department of Gastroenterology and Alimentary Tract Surgery and Tampere Pancreas Laboratory, Tampere University Hospital, Tampere, Finland; 6 Department of Medical Biochemistry, School of Medicine, University of Tampere, Tampere, Finland; Florida International University, United States of America

## Abstract

Cholesterol-lowering treatment has been suggested to delay progression of prostate cancer by decreasing serum LDL. We studied *in vitro* the effect of extracellular LDL-cholesterol on the number of prostate epithelial cells and on the expression of key regulators of cholesterol metabolism. Two normal prostatic epithelial cell lines (P96E, P97E), two *in vitro* immortalized epithelial cell lines (PWR-1E, RWPE-1) and two cancer cell lines (LNCaP and VCaP) were grown in cholesterol-deficient conditions. Cells were treated with 1–50 µg/ml LDL-cholesterol and/or 100 nM simvastatin for seven days. Cell number relative to control was measured with crystal violet staining. Changes in mRNA and protein expression of key effectors in cholesterol metabolism (HMGCR, LDLR, SREBP2 and ABCA1) were measured with RT-PCR and immunoblotting, respectively. LDL increased the relative cell number of prostate cancer cell lines, but reduced the number of normal epithelial cells at high concentrations. Treatment with cholesterol-lowering simvastatin induced up to 90% reduction in relative cell number of normal cell lines but a 15–20% reduction in relative number of cancer cells, an effect accompanied by sharp upregulation of HMGCR and LDLR. These effects were prevented by LDL. Compared to the normal cells, prostate cancer cells showed high expression of cholesterol-producing HMGCR but failed to express the major cholesterol exporter ABCA1. LDL increased relative cell number of cancer cell lines, and these cells were less vulnerable than normal cells to cholesterol-lowering simvastatin treatment. Our study supports the importance of LDL for prostate cancer cells, and suggests that cholesterol metabolism in prostate cancer has been reprogrammed to increased production in order to support rapid cell growth.

## Introduction

Current literature suggests that cholesterol may play an important role in the development and progression of prostate cancer. Several epidemiologic studies have reported a significant positive correlation between hypercholesterolemia or dyslipidemia and prostate cancer incidence [1]–[7]. Experimental studies support these findings, as elevation of circulating cholesterol promotes tumor growth and tumor cholesterol content in a mouse LNCaP xenograft model [8], [9], while reduction in cholesterol levels retards prostate cancer growth, possibly by inhibition of tumor angiogenesis [10]. Recently, epidemiological and laboratory studies have suggested that cholesterol-lowering statin drugs might lower the risk of advanced prostate cancer [11].

*In vitro* studies have proposed that the elevated cholesterol levels in prostate tumor cells could be due to dysregulation of the key regulators of cholesterol homeostasis [12], [13], which could have significance in the progression of prostate cancer into androgen-independent state [14], [15]. Very little is currently known, however, about cholesterol metabolism in normal prostatic epithelial cells and its differences compared to cancer cells.

In the present study we evaluated the effect of cholesterol on growth of both primary and *in vitro* immortalized prostate epithelial cells, and on the growth of androgen-dependent cancer cells. Additionally, we studied the effects of cholesterol and statin treatment on the expression of key participants in cholesterol metabolism: 3-hydroxy-3-methylglutaryl-Coa-reductase (HMGCR), a rate-limiting enzyme in cholesterol-producing mevalonate pathway; Low density lipoprotein receptor (LDLR), required for LDL uptake; Sterol-regulatory element binding protein 2 (SREBP2), regulator of intracellular cholesterol content [16] and the ATP-binding cassette, subfamily A, member 1 (ABCA1), which mediates the efflux of cellular cholesterol [17].

## Materials and Methods

### Materials

Phenol red-free RPMI 1640, fetal calf serum (FCS), L-glutamine, antibiotic-antimycotic solution (A/A), keratinocyte-SFM (K-SFM), recombinant epidermal growth factor (rEGF), and bovine pituitary extract (BPE) were from Invitrogen (Carlsbad, CA, USA). Simvastatin and Low Density Lipoproteins, Human Plasma (LDL) were purchased from Calbiochem (Gibbstown, NJ, USA). Anti-beta-actin antibody (AC-15) was obtained from Sigma (St. Louis, MO, USA). Anti-rabbit IgG, Horse Radish Peroxidase (HRP) –linked antibody and anti-mouse IgG, HRP-linked antibody were from Cell Signaling Technology Inc. (Danvers, MA, USA). Antibody for 3-hydroxy-3-methylglutaryl-CoA reductase (HMGCR (C-1)) was from Santa Cruz Biotechnology, Inc. (Santa Cruz, CA, USA). Antibody for ATP-binding cassette, sub-family A (ABC1), member 1 (ABCA1 (Clone AB.H10)) was from Millipore (Billerica, MA, USA). Antibody for low density lipoprotein receptor (LDLR (EP1553Y)) was from Novus Biologicals, LLC (Littleton, CO, USA) and antibody for Sterol regulatory element-binding protein 2 (SREBP2 (Clone IgG-1C6)) was from BD Biosciences (Franklin Lakes, NJ, USA). Lipoprotein deficient serum (LPDS) was created as described earlier [18]. Corning® Cellbind® 6-well plates were purchased from Corning (Corning, NY, USA). All other disposable cell culture materials were from Nalge Nunc International (Rochester, NY, USA).

### Cell Lines and Culture Conditions

Generation and authentication of P96E and P97E primary prostatic normal epithelial cell lines has been described previously [19]. RWPE-1 and PWR-1E cells (immortalized prostate epithelial cell lines) were a gift from VTT Technical Research Centre, Turku, Finland. P96E, P97E, PWR-1E and RWPE-1 cells were cultured in K-SFM supplemented with 50 µg/ml BPE, 5 ng/ml rEGF and 1% A/A. LNCaP prostate cancer cells were from American Type Culture Collection (Rockville, MD, USA). VCaP prostate cancer cells were a gift from Professor T. Visakorpi, IBT institute, University of Tampere, Finland. LNCaP and VCaP cells were cultured in RPMI 1640 supplemented with 10% FCS, 1% L-glutamine and 1% A/A.

For studies on cell number relative to control, 4×10^4^ (PWR-1E), 5×10^4^ (RWPE-1), 6×10^4^ (P96E, P97E and LNCaP) or 3×10^5^ (VCaP) cells per well were seeded on 6-well plates and allowed to attach for 48 hours. LNCaP and VCaP cells were grown on Corning® Cellbind® 6-well plates, whereas normal cell lines were grown on 6-well plates from Nalge Nunc International. After attachment, LNCaP and VCaP cells were grown in lipid deficient medium (RPMI 1640 supplemented with 10% LPDS, 1% L-glutamine and 1% A/A). The normal prostate epithelial cells were routinely grown in Keratinocyte-SFM which is serum free and essentially lipid deficient.

The cells were treated with LDL-cholesterol or vehicle (DMSO) for seven days. LDL-cholesterol was used in 1–50 µg/ml concentrations to test the dose-dependence of effect. This is the concentration range in standard cell culture conditions when 10% fetal calf serum is being used [20]. This range also allows proper functioning of the LDL-receptor [21]. The highest concentration (50 µg/ml) is in the range of that found in human plasma (from <100 mg/dl to >250 mg/dl) assuming relation 10∶1 between concentration in plasma to that of interstitial tissue.

Growth medium and drugs were renewed every other day. After treatments, the cells were fixed, stained and their number was assessed with modified crystal violet staining method [22]. Absorbances were measured at day 0 and day 7with a Victor 1420 Multilabel Counter (Wallac, Turku, Finland), and the value at day 0 was subtracted from the values at day 7.

For the RNA and protein studies, the cells were seeded to 75 cm^2^ flasks and allowed to attach for 48 hours. After attachment, the cells were grown in lipid deficient medium as described above and treated with vehicle (DMSO), 100 nM simvastatin, 50 µg/ml LDL-cholesterol or in their combination for 48 hours and then subjected to Trizol (Invitrogen, Carlsbad, CA, USA) reagent for RNA extraction or M-PER® (PIERCE, Rockford, IL, USA) reagent modified with protease inhibitors (Complete Mini Protease inhibitor cocktail tablets (Roche Diagnostics GmbH, Indianapolis, IN, USA)) for protein extraction according to the manufacturer’s instructions.

### SDS-PAGE and Western Blot

Total protein concentrations were measured using BCA Protein Assay Kit (Pierce) according to the manufacturer’s instructions. 50 µg of total protein was mixed (1∶1) with 2X Laemmli sample buffer (Sigma, ST. Louis, MO, USA), boiled for 5 min and analyzed by electrophoresis in 12% polyacrylamide gel (PAGE). An exception to this, protein samples for HMGCR were not boiled to avoid protein aggregation upon heating. Precision Plus Protein Standards were used (Bio-Rad Laboratories, Hercules, CA, USA). Proteins separated by PAGE were transferred (1 hour) to the Immobilon-P polyvinylidene fluoride transfer membrane (0.45 µm pore size) (Millipore, Billerica, MA USA) at room temperature (RT) using NuPage transfer buffer (Invitrogen, Carlsbad, CA, USA) according to the manufacturer’s instructions. Membranes were then incubated for 1 hour at RT in Tris buffer containing salt and Tween (TBST) (50 mM Tris-HCL, 150 mM NaCL, 0,05% Tween 20, pH 8.0) and 5% non-fat dry milk powder (5% milk-TBST) to saturate the non-specific protein binding sites. Membranes were incubated with the primary antibodies in 5% milk-TBST overnight at 4°C with mild agitation. The membranes were washed 3 times for 5 min with TBST and incubated for 1 hour with horse radish peroxidase -conjugated secondary antibody in 5% milk-TBST with mild agitation at RT. The membranes were washed 3 times for 5 min with TBST and subjected to enhanced chemiluminescence reagents (ECL Western Blotting Detection Reagents, GE Healthcare, Buckinghamshire, UK) according to the manufacturer’s instructions and exposed to X-ray film.

### Real-Time RT-PCR

The RNA samples were reverse transcribed to cDNA with High Capacity Archive Kit (Applied Biosystems, CA, USA) following the instructions of the manufacturer. The real-time RT-PCR was performed by using SYBR Green PCR Master Mix Kit (Applied Biosystems) in ABI PRISM 7000 Detection System (Applied Biosystems) according to the manufacturer’s instructions. The data were analyzed by ABI PRISM 7000 SDS Software (Applied Biosystems). The final results, expressed as N-fold relative differences (ratio) in gene expression between the studied samples and the control (i.e. calibrator) sample, were calculated according to the following equation [15]: Ratio  =  ((E_target_)^ΔCP target (control-sample)^)/((E_ref_)^ ΔCP ref (control-sample)^). E_target_ is the real-time PCR efficiency of target gene transcript; E_ref_ is the real-time PCR efficiency of a reference gene transcript; ΔCP_target_ is the CP (crossing point) deviation of control – sample (subtraction) of the target gene transcript; ΔCP_ref_ is the CP deviation of control – sample of reference gene (Beta-actin) transcript. Real-time PCR efficiencies (E) were calculated, according to E = 10^[-1/slope]^. Following primers (TAG, Copenhagen, Denmark) were used: HMGCR forward primer (f) 5′- GGC TGC AGA GCA ATA GGT CTT G -3′ and HMGCR reverse primer (r) 5′- CAC GTG GAA GAC GCA CAA CT -3′. LDLR (f) 5′- AGT TGG CTG CGT TAA TGT GAC A -3′ and LDLR (r) 5′- CTC TAG CCA TGT TGC AGA CTT TGT -3′. SREBP2 (f) 5′- CAA GTC TGG CGT TCT GAG GAA -3′ and SREBP2 (r) 5′- GCC CTT TAG AAG CTT GTT CTT TTG -3′. ABCA1 (f) 5′- GAG CAC CAT CCG GCA GAA -3′ and ABCA1 (r) 5′- CTC CGC CTT CAC GTG CTT -3′. Beta-actin (f) 5′-CCA GCT CAC CAT GGA TGA TG -3′ and Beta-actin (r) 5′- ATG CCG GAG CCG TTG TC -3′. The primers were designed using Primer Express software for ABI PRISM 7000 detection system (Applied Biosystems).

### Statistical Analysis

All experiments were repeated separately three times. The median, the highest and the lowest values are reported for each treatment. The non-parametric Mann-Whitney U-test was used to analyze the statistical significance of differences in the outcome measurements between treatments. All p-values are two-sided.

## Results

### LDL, Simvastatin and Cell Number

**Figure 1:**
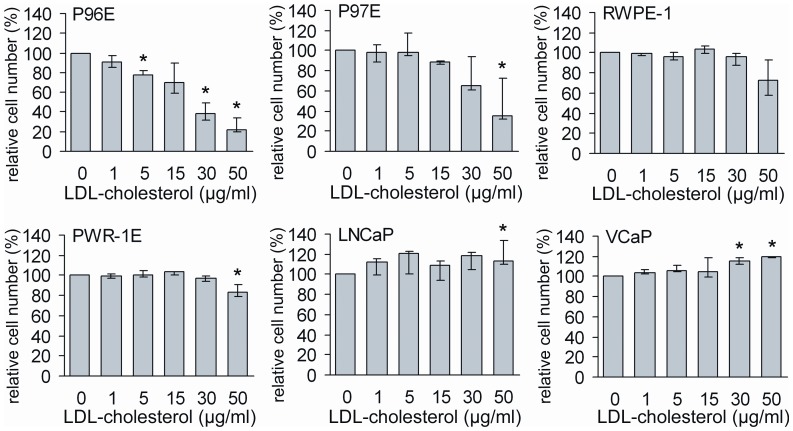
A dose-dependent effect of cholesterol on relative cell number of prostate epithelial cell lines. Number of the treated cells was compared relative to the respective untreated (0) cells after seven days treatment. Results represent the median (bar), lowest and highest (error bars) results of three independent experiments. *p<0.04.

The response in cell number relative to control to increasing concentrations of LDL-cholesterol differed between cancer cells and primary or transformed prostate epithelial cells (Fig.1). High concentrations (30 and 50 µg/ml) of LDL-cholesterol clearly reduced the number of primary cells. However, only slight reduction in the relative cell number was observed in PWR-1E when the highest concentration of LDL-cholesterol (50 µg/ml) was used. On the other hand, relative cell number of both cancer cell lines was slightly stimulated by LDL-cholesterol at the highest concentrations (Fig.1).

**Figure 2:**
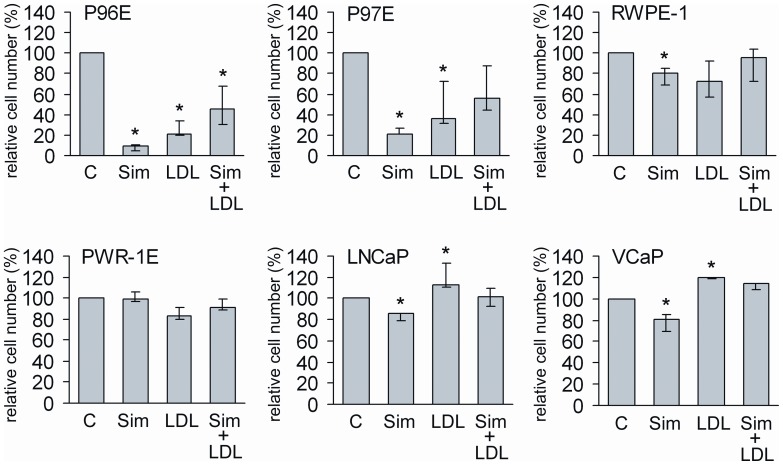
The effect of simvastatin (Sim), LDL-cholesterol (LDL) or combination (Sim + LDL) on cell number relative to control. The cell lines were treated with 100 nM Sim, 50 µg/ml LDL or in combination for seven days. Number of the treated cells was compared relative to the respective untreated control (C) cells. Results represent the median (bar), lowest and highest (error bars) results of three independent experiments. *p<0.04.

Both 100 nM simvastatin and 50 µg/ml LDL-cholesterol reduced the number of normal epithelial cells, with the exception of PWR-1E (Fig. 2). LDL-cholesterol attenuated the relative cell number reduction caused by simvastatin in P96E and P97E cells (p<0.05 for difference between combination of simvastatin and LDL treatment as compared to simvastatin alone), although the reduction relative to control cells remained significant. Compared to the control, addition of LDL to simvastatin removed the significant relative cell number decreasing effect of simvastatin alone in RWPE-1 cells, although the difference between the two treatments remained non-significant. In cancer cell lines simvastatin caused only modest reduction in relative cell number, an effect fully compensated by LDL-cholesterol (Fig 2). Simvastatin slightly reduced the relative cell number increasing effect of LDL-cholesterol on cancer cells.

### Expression of Cholesterol Metabolizing Factors at Baseline

**Figure 3:**
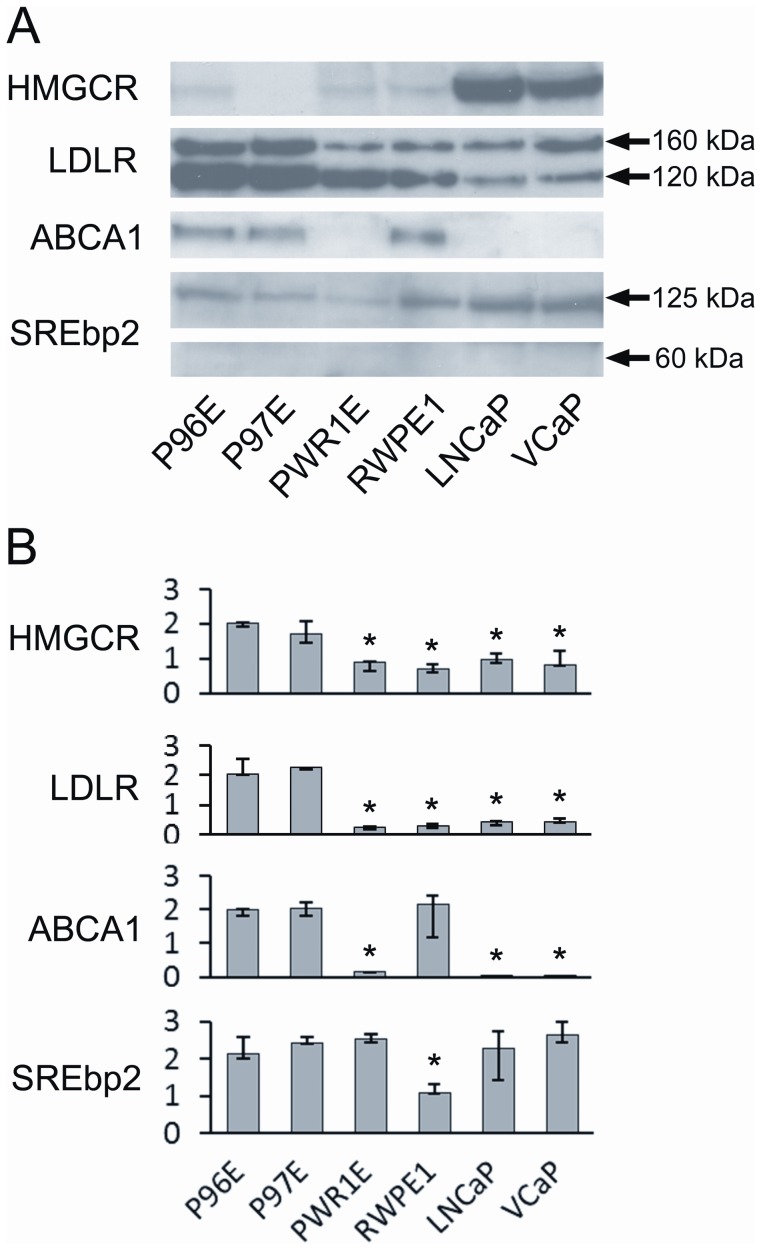
Basal protein expression (a) and mRNA expression (b) of HMG-CR, LDLR, ABCA1 and SREBP2 in prostate epithelial cell lines. All cell lines were grown in lipid-deficient medium. A protein band of 90 kilodaltons (kDa) is shown for HMG-CR and a 254 kDa band for ABCA1. A double band of 120 and 100 kDa is shown (arrows) for LDLR. For SREBP2, the 125 kDa precursor and 60 kDa cleaved mature form is shown (arrows). *p<0.04.

The basal protein expression levels of important regulators of cellular cholesterol metabolism in a standard amount of protein were compared between normal epithelial cells and cancer cell lines after the cells had been grown in cholesterol-deficient medium for seven days. All cell lines expressed SREBP2 at protein level, cancer cell lines more strongly than normal cell lines (Fig. 3a, suppl. Fig S1), although the mRNA expression did not differ greatly between the cell lines (Fig 3b). The exception was RWPE-1, where mRNA expression of SREBP2 was low compared to any other cell line.

Under these circumstances the cancer cell lines exhibited upregulation of HMGCR at protein level, suggesting increased cholesterol production, whereas normal primary cells showed upregulation of LDLR (Fig 3a, suppl. Fig S1). Again, baseline mRNA expression differed from protein expression as both HMGCR and LDLR expression were markedly higher in normal primary cells P96E and P97E as compared to cancer cell lines (Fig 3b). In PWR-1e and RWPE-1 the mRNA expressions were similar to cancer cell lines.

Even under depletion of extracellular cholesterol, the normal epithelial cells (with the exception of PWR-1E) expressed cholesterol transporter ABCA1 at protein level, whereas cancer cell lines did not (Fig. 3a, suppl. Fig S1). For ABCA1 The mRNA expression was similar to protein expression: high expression in normal cell lines P96E, P97E and RWPE-1, but almost no expression in cancer cell lines and PWR-1e (Fig 3b).

### Effect of LDL and Simvastatin on the Expression of Key Cholesterol-metabolizing Factors

**Figure 4:**
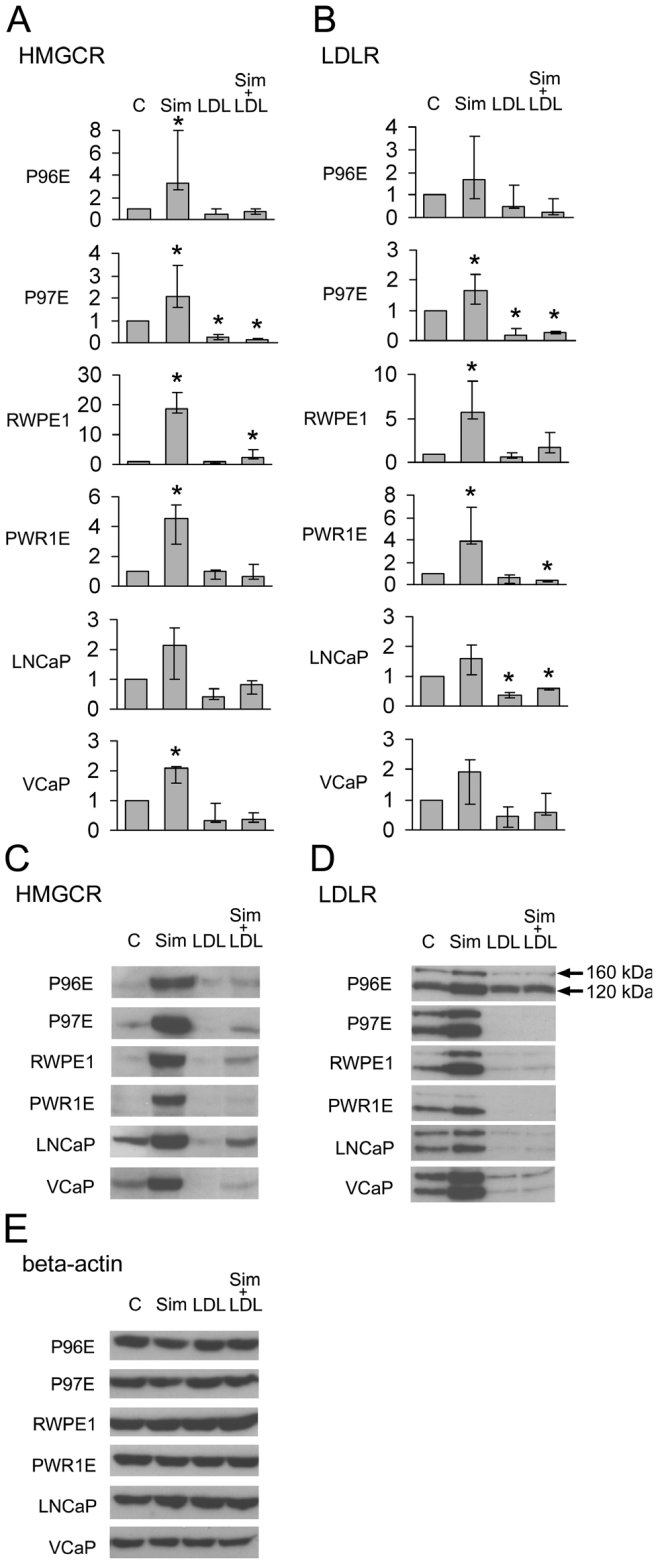
Analysis of HMG-CR and LDLR in prostate epithelial cell lines. The mRNA levels measured by RT-PCR (a and b) and protein levels by Western blotting (c and d). Beta-actin expression is reported in e). The cell lines were treated with DMSO (C) 100 nM Sim (Sim), 50 µg/ml LDL-cholesterol (LDL) or in combination (Sim + LDL) for 48 hours. A double band of 120 and 100 kilodaltons (kDa) is shown (arrows) for LDLR and a 90 kDa band for HMG-CR. The HMG-CR and LDLR mRNA expression levels were calculated relative to the DMSO-treated (C) samples. RT-PCR results represent the median (bar), lowest and highest (error bars) results of three independent experiments. *p<0.04.

**Figure 5:**
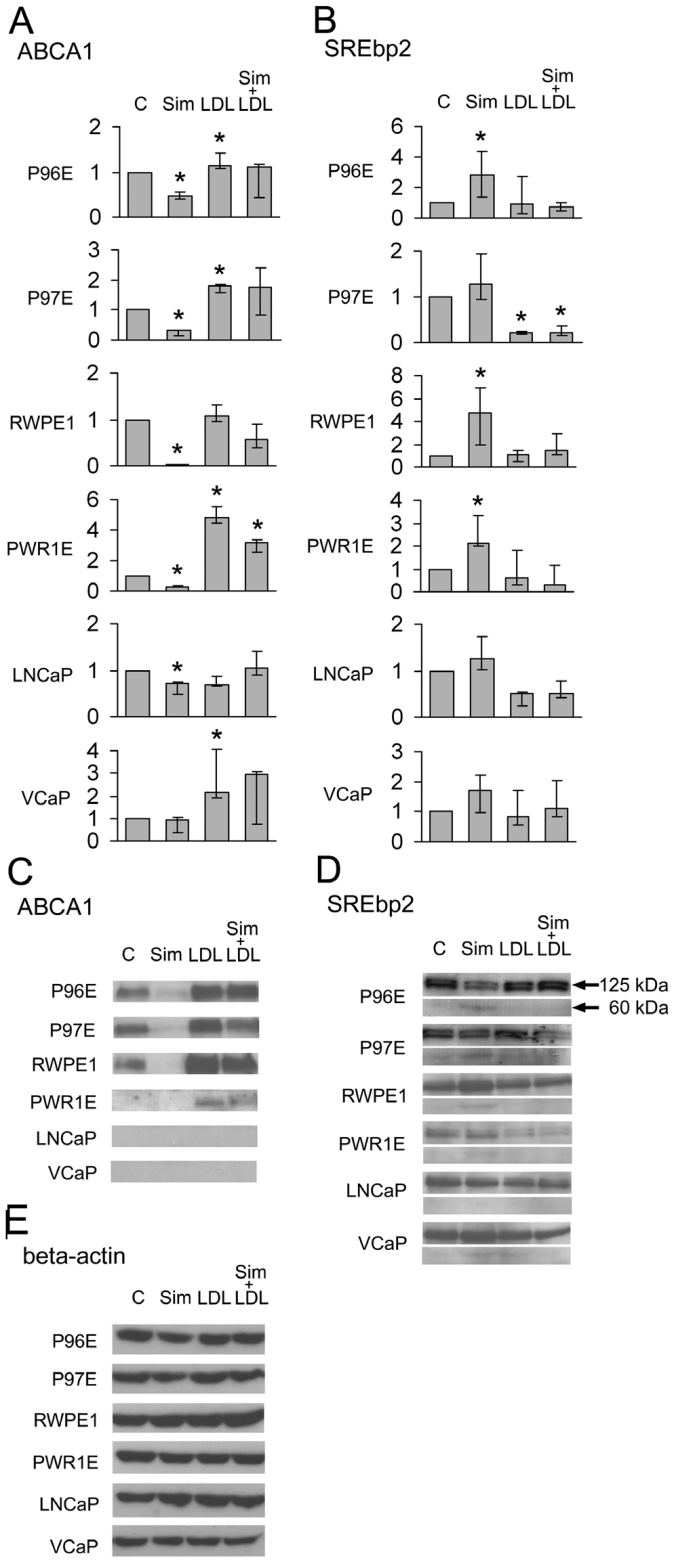
Analysis of ABCA1 and SREBP2 in prostate epithelial cell lines. The mRNA levels measured by RT-PCR (a and b) and protein levels by Western blotting (c and d). Beta-actin expression is reported in e). The cell lines were treated with DMSO (C) 100 nM Sim (Sim), 50 µg/ml LDL-cholesterol (LDL) or in combination (Sim + LDL) for 48 hours. A double band of 125 kilodaltons (kDa) (precursor form) and 60 kDa (cleaved form) is shown (arrows) for SREBP2 and a 254 kDa band for ABCA1. The ABCA1 and SREBP2 mRNA expression levels were calculated relative to the DMSO-treated (C) samples. RT-PCR results represent the median (bar), lowest and highest (error bars) results of three independent experiments. *p<0.04.

Inhibition of *de novo* cholesterol synthesis with simvastatin sharply upregulated the mRNA (Fig. 4 a and b) and protein expressions of HMGCR and LDLR (Fig. 4c and d) in all cell lines. Simvastatin also upregulated mRNA expression of SREBP2 (Fig. 5b). At protein level simvastatin treatment did not increase SREBP2 expression, but rather caused cleavage of the protein into 125 kDa and 60 kDa bands (Fig. 5d). In normal cells the expression of ABCA1 was clearly down-regulated by simvastatin (Fig. 5a and c). In cancer cells simvastatin did not markedly affect ABCA1 expression (Fig. 5a and c).

Compared to control, LDL-cholesterol downregulated HMGCR mRNA expression significantly only in P97E, while downregulation of protein expression was most clearly observed in cancer cell lines LNCaP and VCaP (Fig 4 a and c). LDL also downregulated LDLR mRNA expression in P97E and LNCaP (Fig 4b), but protein expression was downregulated in all cell lines except P96E (Fig. 4d). The response in ABCA1 differed between cancer cells and normal cells: availability of extracellular LDL upregulated ABCA1 in the normal cell lines, but the cancer cells did not express this transporter at detectable protein level even after LDL-cholesterol treatment, although slight changes were observed in mRNA expression (Fig. 5a and c). LDL decreased the mRNA expression of SREBP2 in P97E, but in other cell lines the expression was comparable to vehicle-treated cells (Fig. 5b). At protein level LDL prevented the effect of simvastatin on cleavage of SREBP2 into two bands in P96E, P97E and RWPE-1 (Fig 5d).

LDL prevented most of the effects of simvastatin on the expression of cholesterol metabolizing factors (Fig. 4a-d and Fig 5a-d). An exception was HMGCR in the LNCaP, where simvastatin caused upregulation of the enzyme expression even in the presence of LDL (Fig. 4a and c).

## Discussion

Our observations support the importance of cholesterol for the growth of prostate cancer cell lines: 1) increase in cell number relative to control after treatment with increasing concentrations of LDL; 2) decreased relative cell number after inhibition of intracellular cholesterol synthesis with simvastatin, which could be prevented by addition of LDL; 3) enhanced expression of HMG-CoA reductase, the rate-limiting enzyme of cholesterol biosynhesis at baseline in cancer cell lines and 4) no evidence of ABCA1 expression in cancer cells under any circumstances, even after LDL treatment.

Cholesterol is important for cell membrane integrity and cellular metabolism, as well as for signalling pathways essential for cellular proliferation, such as PI3K/Akt [23]. Combined, our results suggest that LDL is needed for growth of prostate cancer cells. Increased expression of the biosynthetic machinery along with no expression of the major participant in cholesterol efflux from the cells suggests reprogramming of cholesterol metabolism in cancer cells. Although remaining responsive to changes in extracellular conditions such as treatment with simvastatin or LDL, the metabolism has been geared towards providing the cells with maximal supply of cholesterol to enable rapid cell growth under any conditions. Even in cholesterol-free conditions inhibition of intracellular cholesterol synthesis with simvastatin reduced the number of cancer cells 15–20%, but up to 90% of normal epithelial cells; presumably higher baseline cholesterol synthesis protects cancer cells against the effects of simvastatin. However, we did not directly measure intracellular cholesterol synthesis.

Besides cholesterol, mevalonate pathway produces also isoprenoids farnesylpyrophosphate (FPP) and geranylgeranylpyrophosphate (GGPP), which in turn have important cell growth regulatory functions [24]. Inhibition of these end-products of mevalonate pathway and resulting cellular changes are termed pleiotropic effects of statins. The differing ability of LDL to restore the relative cell number reduction caused by simvastatin between the cell lines could have been due to differing role of pleiotropic effects. In future the relationship between inhibition of isoprenoid production and cholesterol production when studying statins’ effects on cell growth should be further studied.

The relative cell number of normal epithelial cell lines was not induced by LDL, but conversely high concentrations caused reduction. Normal cells also require cholesterol for cell growth as treatment with simvastatin caused a powerful growth inhibition, again restored by addition of LDL. Normal cells responded to simvastatin treatment by increasing HMGCR and LDLR expressions, but unlike the cancer cell lines, normal cells also increased expression of cholesterol exporting transporter ABCA1 as a result of treatment with LDL. This suggests that normal cells need equilibrium in cholesterol homeostasis for undisturbed cell growth. The changes in normal cells reflect attempts to adapt to changing extracellular conditions by adjusting intracellular cholesterol metabolism to maintain the equilibrium. Very high LDL concentrations, however, likely exceed this adaptive potential, causing toxic growth inhibition. Such was not observed in cancer cell lines, however. These differences between normal prostatic epithelial cells and cancer cell lines reflect the changes in cholesterol metabolism occurring during carcinogenesis in the prostate. Likely reprogramming of cholesterol metabolism is a crucial part of the rearrangement of energy metabolism in cancer cells supporting constant proliferation [25], a trait that has been recognized as one of the hallmarks of cancer [26].

Also in previous studies cholesterol has increased the growth of prostate cancer cell lines PC3 and DU-145 [27], [28]. Unlike in our study LDL treatment has not been previously found to induce growth of LNCaP cells [29], despite similar downregulation of LDLR expression. The discrepancy in the results is possibly explained by the shorter duration of LDL treatment in the previous study (48 h) compared to ours (seven days). In this paper we focused on effects of LDL to further explore the association with prostate cancer risk reported in epidemiological studies [7], [30] and observed in our previous studies [31]. Nevertheless, also high-density lipoprotein (HDL) has been reported to induce prostate cancer cell growth [32], suggesting that cancer cells can probably use various types of lipoproteins as a source of cholesterol.

The importance of cholesterol for prostate cancer growth is further supported by experimental studies, where elevation of circulating cholesterol has been reported to increase tumor growth and intra-tumoral cholesterol accumulation in a mouse LNCaP xenograft model [8], [9], PC-3 xenograft [27] and DU-145 xenograft [33]. A hypercholesterolemic diet changes prostate morphology in male Wistar rats [34]. On the other hand, reducing cholesterol levels retards prostate cancer growth possibly by inhibition of tumor angiogenesis in a prostate cancer xenograft model [10].

Changes in the expression levels of the key regulators of cholesterol homeostasis, namely sterol regulatory element binding transcription factors (SREBPs), HMGCR LDLR, acetyl-CoA acetyltransferase 1 (ACAT1) and scavenger receptor class B member 1 (SR-B1) have been shown to occur during the progression of prostate cancer from androgen-independent to castration-resistant cancer in an LNCaP xenograft model [14], [15]. Cholesterol influx by SR-B1 is essential for viability of prostate cancer cell lines such as LNCaP [35]. We have demonstrated that marked differences in expression of key regulators of cholesterol metabolism are observed already between normal epithelial cell lines and androgen responsive LNCaP and VCaP cancer cell lines. Nevertheless, cholesterol metabolism remains responsive to extracellular stimuli; our results are in concordance with a previous study by Krycer et al [13] reporting feedback regulation of SREBP2, HMGCR and LDLR mRNA expression in cancer cells and normal epithelial cells by extracellular cholesterol. We further show that this regulation occurs at protein level, and also in primary normal prostate epithelial cells which have been isolated directly from prostatic tissues. The differences observed between mRNA and protein expressions of HMGCR, LDLR and SREBP2 suggests that mRNA of these enzymes may undergo posttranslational modifications before transcription into protein level. Further research will be needed.

In vivo evidence for the importance of cholesterol in prostate cancer progression comes from epidemiological studies reporting increased risk of advanced prostate cancer among hypercholesterolemic men [1], [2]. Serum cholesterol decreases spontaneously within nine years before a cancer diagnosis [3], which might indicate that a developing tumor consumes cholesterol from the circulation to enable cell growth; a notion supported by some *in vitro* studies [36]. Improved recurrence-free survival after radical treatment of prostate cancer has been reported among men using cholesterol-lowering statin drugs, an association possibly related to serum LDL decrease during statin therapy [37].

We could not test the responses of early-stage prostate cancer cells to LDL and statin treatments as these are not currently commercially available. However, it could be reasonably presumed that the responses of well-differentiated prostate cancer cells at the early stages of carcinogenesis resemble those of normal epithelial cells. In our study the cells were grown in monolayer cultures, whereas *in vivo* prostate epithelial cells are in close contact with the surrounding stroma, which has important functions in carcinogenesis [26] and could modify epithelial cells’ responses to LDL and simvastatin. Thus *in vivo* studies will be needed to confirm our findings.

We have shown that increasing doses of LDL induce number of prostate cancer cells, but not normal epithelial cells. Both normal and cancer cells increase the production of effectors that ensure the synthesis and uptake of cholesterol under depletion, but cancer cells do not express the major exporter of cholesterol, ABCA1 even in the abundance of LDL. Cholesterol availability is likely an important prerequisite for prostate cancer growth and cholesterol metabolism in prostate cancer cells is reprogrammed to supply the cells with abundance of cholesterol. Cholesterol-lowering might prove to be a good strategy to prevent and delay prostate cancer progression. Hypercholesterolemia as an etiologic factor for prostate cancer deserves further studies.

## Supporting Information

Figure S1 **Quantification of relative intensities of immunoblotted bands shown in** **Fig 3** **.** Relative intensities of bands on Western blots were quantified using ImageJ 1.45 (http://imagej.nih.gov/ij/ *)* according to instructions by Luke Miller available in http://lukemiller.org/index.php/2010/11/analyzing-gels-and-western-blots-with-image-j/with minor modifications. Shortly, band density for a given protein in different cell types was divided with that of P96E cells, to obtain relative densities of bands. The relative densities in P96E cells represent the value 1. Values below 0.1 are denoted <0.1. Cases in which no band was detected are denoted as n.d. (not detected).
(DOC)
