# Supplementary material for: The Importance of LDL and Cholesterol Metabolism for Prostate Epithelial Cell Growth
Source: PLoS One. 2012 Jun 27;7(6):e39445. doi: 10.1371/journal.pone.0039445 (PMC3384647; doi:10.1371/journal.pone.0039445)
Supplement: Figure S1 — Quantification of relative intensities of immunoblotted bands shown in Fig 3 . Relative intensities of bands on Western blots were quantified using ImageJ 1.45 (http://imagej.nih.gov/ij/ ) according to instructions by Luke Miller available in http://lukemiller.org/index.php/2010/11/analyzing-gels-and-western-blots-with-image-j/with minor modifications. Shortly, band density for a given protein in different cell types was divided with that of P96E cells, to obtain relative densities of bands. The relative densities in P96E cells represent the value 1. Values below 0.1 are denoted <0.1. Cases in which no band was detected are denoted as n.d. (not detected). (DOC) [file pone.0039445.s001.doc]

**Supplemental Figure S1**

|  | **HMGCR**  (rel.density) | **LDLR**  (**160kD**)  (rel.density) | **LDLR** (**120kD**)  (rel.density) | **ABCA1**  (rel.density) | **SREbp-2**  (**125kD**)  (rel.density) | **SREbp-2**  ( **60kD**)  (rel.density) |
| --- | --- | --- | --- | --- | --- | --- |
| P96E | 1 | 1 | 1 | 1 | 1 | n.d. |
| P97E | <0.1 | 1.0 | 0.9 | 0.9 | 0.5 | n.d. |
| PWR-1E | 0.7 | 0.3 | 0.6 | n.d. | 0.3 | n.d. |
| RWPE-1 | 1.0 | 0.4 | 0.5 | 1.1 | 1.1 | n.d. |
| LNCaP | 9.5 | 0.4 | 0.2 | n.d. | 1.8 | n.d. |
| VCaP | 8.0 | 0.7 | 0.3 | n.d. | 1.9 | n.d. |
